# The Impact of Gut Microbiota Changes on Methotrexate-Induced Neurotoxicity in Developing Young Rats

**DOI:** 10.3390/biomedicines12040908

**Published:** 2024-04-19

**Authors:** Yu-Chieh Chen, Chih-Yao Hou, Mei-Hsin Hsu, Li-Tung Huang, Chih-Cheng Hsiao, Jiunn-Ming Sheen

**Affiliations:** 1 Department of Pediatrics, Kaohsiung Chang Gung Memorial Hospital, Kaohsiung 833, Taiwan; 2 Department of Traditional Medicine, Chang Gung University, Taoyuan 333, Taiwan; 3 School of Medicine, College of Medicine, National Sun Yat-sen University, Kaohsiung 804, Taiwan; 4 Department of Seafood Science, College of Hydrosphere, National Kaohsiung University of Science and Technology, Kaohsiung 807, Taiwan

**Keywords:** methotrexate, chemotherapy, cognitive impairment, gut microbiota, neurotoxicity

## Abstract

Methotrexate (MTX) is an essential part of therapy in the treatment of acute lymphoblastic leukemia (ALL) in children, and inferior intellectual outcomes have been reported in children who are leukemia survivors. Although several studies have demonstrated that the interaction between gut microbiota changes and the brain plays a vital role in the pathogenesis of chemotherapy-induced brain injury, preexisting studies on the effect of MTX on gut microbiota changes focused on gastrointestinal toxicity only. Based on our previous studies, which revealed that MTX treatment resulted in inferior neurocognitive function in developing young rats, we built a young rat model mimicking MTX treatment in a child ALL protocol, trying to investigate the interactions between the gut and brain in response to MTX treatment. We found an association between gut microbiota changes and neurogenesis/repair processes in response to MTX treatment, which suggest that MTX treatment results in gut dysbiosis, which is considered to be related to MTX neurotoxicity through an alteration in gut–brain axis communication.

## 1. Introduction

With improvements in diagnostic and therapeutic strategies, the long-term survival rate of childhood acute lymphoblastic leukemia (ALL) has reached approximately 90% [1,2,3]. However, follow-up studies on childhood leukemia survivors have revealed impaired intellectual and cognitive functions compared with age-matched children [4,5,6,7,8], which has sparked research interest about the neurologic impact of chemotherapy medications on ALL survivors. Among the medications commonly reported to be related to late cognitive sequelae in childhood ALL survivors, methotrexate (MTX) has been widely reported to be related to cognitive impairment in children, and it is either administered by intravenous (IV) or intrathecal (IT) routes [9]. Clinical studies have shown that MTX chemotherapy, especially combined IT and IV treatment, is responsible for both functional and morphological changes in the brain and can result in serious late neurologic sequelae, notably cognitive impairment [10,11]. Recently, Wen et al. used combined IP and IT MTX protocols and found impaired memory, neuroinflammation, and neurogenesis in developing rats [12]. While most preclinical studies have focused on MTX treatment in adult rodents, studies focused on developmental young rats are lacking. We found that combined IP and IT MTX treatment caused spatial memory deficits in developing rats, using an animal model mimicking the treatment protocol for children with ALL, and using young rats treated with IP, IT, or combined IT and IP MTX [13]. In addition, we demonstrated that the loss of adaptive myelination contributes to MTX neurotoxicity [14]. In this study, we plan to further explore the impact of MTX on gut microbiota changes, based on our previous work, in order to understand the interactions between the gut and brain following MTX treatment in developing young rats.

Although gut microbiota changes following MTX treatment have been reported in the literature [15,16], most studies have focused on microbiota alterations and gastrointestinal (GI) toxicity following MTX treatment [17,18]. The impact of gut microbiota changes following MTX treatment on neurocognitive function has not yet been reported. Moreover, the effect of MTX on the developing subjects, with regard to the interaction between the gut and brain, has not been mentioned in the literature. Since increasing studies have documented the close relationship between gut microbiota change and various neurologic disorders [19,20,21,22], and growing studies indicate a close relationship between gut microbiota changes and chemobrain [23], our present study was designed to evaluate the impact of MTX on gut microbiota changes and its relationship to MTX-induced neurocognitive impairment, specifically focusing on the developing young rats. Also, this study, as an extension of our previous work with an emphasis on the elucidation of the role of gut microbiota change in the pathogenesis of MTX neurotoxicity, serves a model to evaluate the gut–brain interaction in MTX-treated young rats. To our knowledge, this is the first study to comprehensively evaluate the alteration in gut microbiota and metabolite changes following MTX treatment, with an emphasis on the impact of MTX on the central nervous system in developing rats.

## 2. Materials and Methods

### 2.1. Experimental Model and Subject Details

The experiments in this study were performed in accordance with the Guidelines for Animal Experiments of the Chang Gung Memorial Hospital and Chang Gung University. The experiments were approved by the Institutional Animal Care and Use Committee, Taiwan (approval number: 2020032404; approval date: 23 April 2020). The day of delivery was defined as Day 0. Male Sprague-Dawley rats (PND 21 ± 1) weighing ~50 g were used, and attempts were made to minimize the number of animals included. All animals were housed in a room maintained at 24 °C with 12 h light/dark cycles. All animals had free access to standard food.

All surgical procedures were performed under Zoletil50 (25 mg/kg) and xylazine (23 mg/kg) anesthesia using clean surgical techniques, as previously described [24] (Huang et al., 2010). Each group of rats received an IT injection via a transcutaneous cisternal magna puncture. Briefly, the rats were anesthetized and placed in the lateral decubitus position. A 27-gauge needle was inserted into the cisterna magna. The correct position was verified by the outflow of cerebral spinal fluid (CSF), and polyethylene catheters were inserted through a small incision in the atlanto-occipital membrane and passed 1.5 cm caudally to the level of the lumbar enlargement of the catheters [25,26]. To confirm the correct placement of the catheters, 10 μL of 0.9% saline was flushed the day after the surgery. Since this is an extension of our previous work based on the finding that MTX treatment results in spatial memory changes in young rats [13,14] and focuses on the gut microbiota change following combined IT and IP MTX treatment, the study animals were divided into two groups, as illustrated in the following, to minimize animal numbers:

1. CTP: rats that underwent catheter implantation surgery and received an equal volume of normal saline to the MTX treatment group via IT and intraperitoneal injection (IP).
2. XTP: rats that underwent catheter implantation surgery and received 0.5 mg/kg of methotrexate diluted with 0.9% normal saline in 5~10 μL as the final volume via IT once per week for two weeks. Then, 24 h after IT injection, rats received 100 mg/kg of methotrexate via IP injection once per week for two weeks.

**Figure 1:**
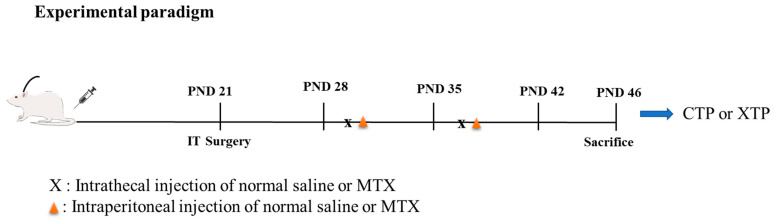
Experimental paradigm.

The detail of the experimental protocol was illustrated as Figure 1.

### 2.2. 16S Metagenomics Studies Using 16S rDNA Next Generation Sequencing (NGS) for Gut-Microbiota Measurements

Fecal samples were collected from rats that received either normal saline (CTP, *n* = 10) or MTX (XTP, *n* = 12). And the fecal genomic DNA was extracted by Quick-DNA Fecal/Soil Microbe Miniprep Kit (ZYMO Research, Irvine, CA, USA). A total of 12.5 ng of gDNA was used for PCR amplification, with universe primers of 16S rRNA for V3eV4 regions (341F: 5′-CCTACGGGNGGCWGCAG-3′, 806R: 5′-GACTACHVGGGTAT CTAATCC-3′), and then sequenced as per the following procedures. Sequence analysis was performed with the Illumina MiSeq (Illumina, San Diego, CA, USA) paired-end sequencing platform. Briefly, 300 bp paired-end reads were assembled by FLASH v1.2.11 from the 16S PCR raw reads [27]. QIIME v1.9.1 pipeline [28] was used to discard low-quality raw reads (Q < 20) [29] and chimera-checked sequences, via UCHIME [30,31] examination, were used to acquire effective tags and were removed from the dataset before the operational taxonomic unit (OTU) clustering at 97% sequence identity using the UPARSE [32] function in the USEARCH v7.0.1090 pipeline [33]. To annotate taxonomy classification in each OTU, the RDP classifier (v2.2) algorithm [34] was used in the process and the following taxonomic assignments were accomplished by comparing them to the Silva Database v132 [35,36] based on an 80% minimum confidence threshold. To maintain the accuracy of the analysis, sequences that were singletons (one-time occurrence) or recognized in only one sample were excluded. Sequence similarity analysis among different OTUs was performed by PyNAST v.1.2 [28] software against the core-set dataset in the SILVA database and a phylogenetic tree was constructed with a set of sequences representative of the OTUs via FastTree [37,38].

The normalization of variations in sequence depth across samples was accomplished using the QIIME script (single_rarefaction.py) to minimize sequence depth in that OTU abundance information was rarefied and then used to evaluate species complexity within individual samples (alpha diversity) and the differences among samples (beta diversity). For alpha diversity, Chao 1 estimator and Shannon index [39], performed by QIIME pipeline, were used in this study; for beta diversity, unweighted UniFrac performed by QIIME pipeline and principal coordinate analysis (PCoA) visualized the sophisticated and multidimensional data by calculating the distance matrix to acquire principal coordinates, which were employed here [40]. Bioinformatics analysis for functional gene profiles was accomplished by enriching the metabolic pathway at level 2 and 3 categories by PICRUSt (v1.1.1) [41] to reveal the influence of MTX in the overall metabolic pathway and by mapping Tax4Fun [42] level 4 genes in KEGG pathways to annotate potential genes in response to MTX.

### 2.3. Brain Tissue Collection

We examined two regions that are involved in spatial performance in rats: the prefrontal cortex and the hippocampus [43]. The prefrontal cortex and hippocampus were removed immediately after the rats were sacrificed, and the tissues were homogenized.

### 2.4. Quantitative Real-Time Polymerase Chain Reaction (PCR) Analysis

PCR analysis was performed as previously reported [24] (Huang et al., 2010). SCFA pathway genes and neurotransmitter-related genes were verified. The gene sequences are shown in the Supplementary Materials.

### 2.5. Short-Chain Fatty Acid Analysis

Gas chromatography–mass spectrometry (GCMS-QP2010; Shimadzu, Kyoto, Japan) with a flame ionization detector (FID) was used to detect the concentrations of acetate, butyrate, and propionate in the plasma. According to Hsu et al., in brief, internal standards in analytical standard grades for acetate, propionate (Sigma-Aldrich, St. Louis, MO, USA), and butyrate (Chem Service, West Chester, PA, USA) were used [44]. All solutions, including the internal and external standards, were prepared at a concentration of 10 mM and stored at −20 °C until use. Dry air, nitrogen, and hydrogen were supplied to the FID at 300, 20, and 30 mL/min, respectively. A 2 µL aliquot of the sample was injected into the column. The inlet and FID temperatures were set to 200 and 240 °C, respectively. The total running time was 17.5 min.

### 2.6. Hippocampus Metabolite Analysis

Hippocampus tissues were weighed and extracted for the neurotransmitter metabolite analysis. Briefly, 80 μL of extract solvent (precooled at −20 °C, acetonitrile, within 0.1% FA) and 20 μL of H_2_O were added into each sample and then vortexed for 30 s, homogenized at 45 Hz for 4 min, and sonicated for 5 min in an ice-water bath multiple times, followed by storage at −40 °C overnight and centrifuging at 12,000 rpm and 4 °C for 15 min. Then, 80 μL of the supernatants was incubated for 30 min after the addition of 40 μL 100 mmol/L sodium carbonate solution and 40 μL 2% benzoyl chloride acetonitrile solution. Next, 10 μL internal standard was added and centrifuged at 12,000 rpm for 15 min at 4 °C. The 40 μL sample of the supernatants was added to 20 μL H_2_O and then transferred to an auto-sampler vial for UHPLC-MS/MS analysis [45].

The separation condition of UHPLC was performed by the ExionLC System (SCIEX ExionLC; SCIEX, Framingham, MA, USA), equipped with a Waters ACQUITY UPLC HSS T3 (100 × 2.1 mm, 1.8 μm). Mobile phase A was 0.1% formic acid and 1mM/L ammonium acetate in water, and mobile phase B was acetonitrile. The column temperature was set at 40 °C. The auto-sampler temperature was set at 4 °C and the injection volume was 1 μL. Mass spectrometric detection was carried out using the AB Sciex QTrap 6500+ mass spectrometer (SCIEX, Framingham, MA, USA), which was applied for assay development. Ion source parameters are listed as follows: IonSpray Voltage: +5000 V; Curtain Gas: 35 psi; Temperature: 400 °C; Ion Source Gas 1: 60 psi; Ion Source Gas 2: 60 psi. The multiple reaction monitoring (MRM) data were analyzed by Skyline Software (v. 1.5.2) [46,47].

### 2.7. Prediction of Metabolic Pathway and Metabolite Changes Following MTX Treatment

PICRUSt v.1.0.0 was performed to predict the alteration of metabolic pathways induced by MTX treatment, and subsequent KEGG orthology (KO) enrichment was merged into hierarchical categories (level 1, level 2, and level 3). In addition, to predict the possible metabolites altered by MTX treatment, Tax4Fun (an open-source R package applicable to output as obtained from the SILVAngs web server or the application of QIIME with a SILVA database) level 4 annotation genes were used and mapped with the KEGG pathway mapper website (https://www.genome.jp/kegg/mapper/search.html, accessed on 22 July 2022), using a search tool and sorting data according to their hierarchy.

### 2.8. Statistical Analysis

Biochemical parameters, ELISA, Western blots, PCR, and neurotransmitter metabolites were analyzed by an independent *t*-test. All analyses were performed using SPSS (Version 20) on a PC-compatible computer. Values were expressed as mean ± SEM and significances were defined as *p* < 0.05 for all tests.

For 16S rDNA sequence data, a zero-inflated Gaussian (ZIG) log-normal model, as implemented in the “fitFeatureModel” function of the Bioconductor metagenomeSeq package [48], was used to examine the significant differential abundance of species at various taxonomic levels. Using STAMP v.2.1.3 [49] software, an unequal variances *t*-test (Welch’s *t*-test) was performed. Linear discriminant analysis effect size (LEfSe, run on R studio (version 3.1.3) with microeco packages) analysis was used for discriminating significant biomarkers using the Mann–Whitney U test to identify significant changes in bacterial taxa between groups and the threshold of LDA score (log_10_) > 3.0 was considered significant.

## 3. Results

### 3.1. MTX Treatment Induced Gut Microbiota Dysbiosis

**Figure 2:**
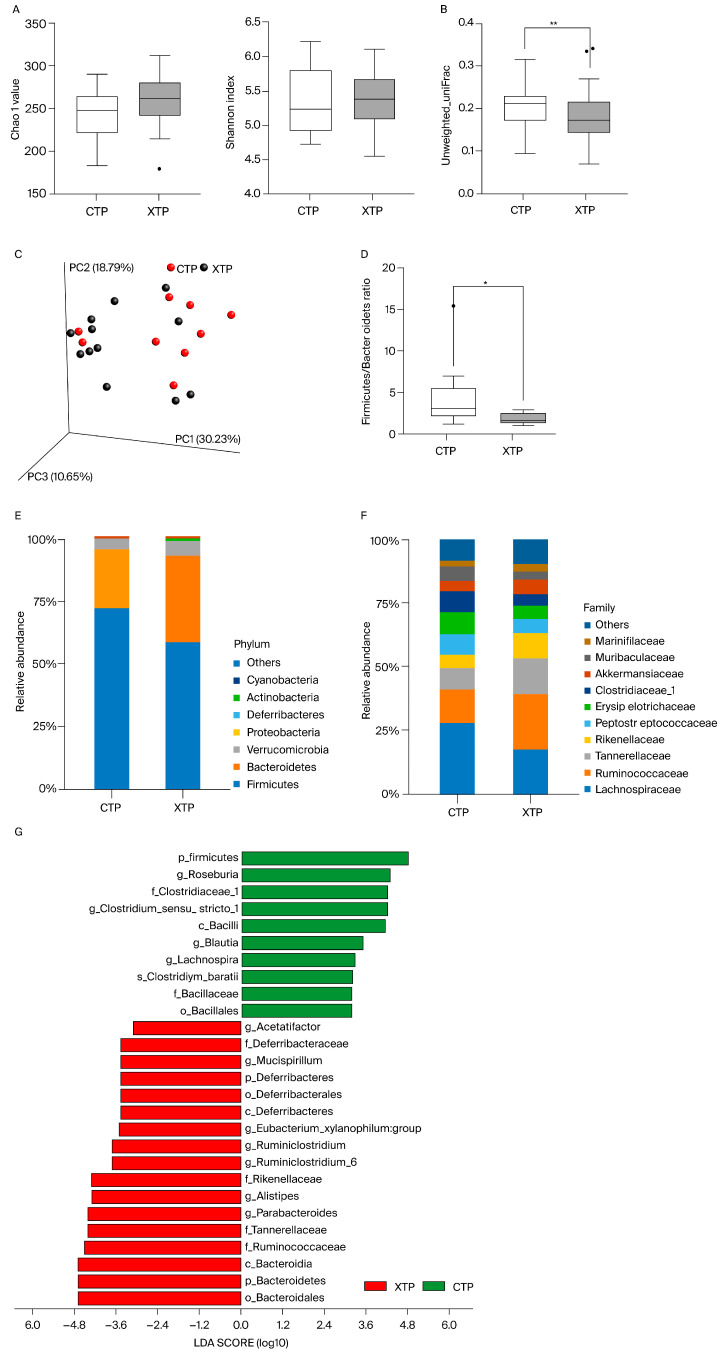
(**A**) Alpha diversity index, measured by Chao1 richness estimator, and Shannon index were not significantly changed. (**B**) Beta diversity index measured by unweighted unifraction distance revealed that local species within groups were changed by MTX treatment. (**C**) PCoA coordinate matrix analysis demonstrated that local species within groups were changed by MTX treatment. (**D**) Firmicutes/Bacteroidetes ratio was decreased in the MTX-treated group. (**E**) Relative abundances of the top 10 clusters of bacteria at the phylum level were changed in the MTX-treated group. (**F**) Relative abundances of the top 10 clusters of bacteria at the family level were changed in the MTX-treated group (values were shown as percentage). (**G**) Linear discriminant analysis (LDA) effect size (LEfSe) analysis found change in bacteria at the genus level after MTX stimulation. (green bar: taxa found in greater relative abundance in the CTP group; red bars: taxa found in greater relative abundance in XTP). LDA scores higher than 3 were shown with significant changes. CTP: sham groups; XTP: MTX-treated groups. *: *p* < 0.05 vs. CTP; **: *p* < 0.01 vs. CTP. N = 10 in CTP; N = 12 in XTP.

**Figure 3:**
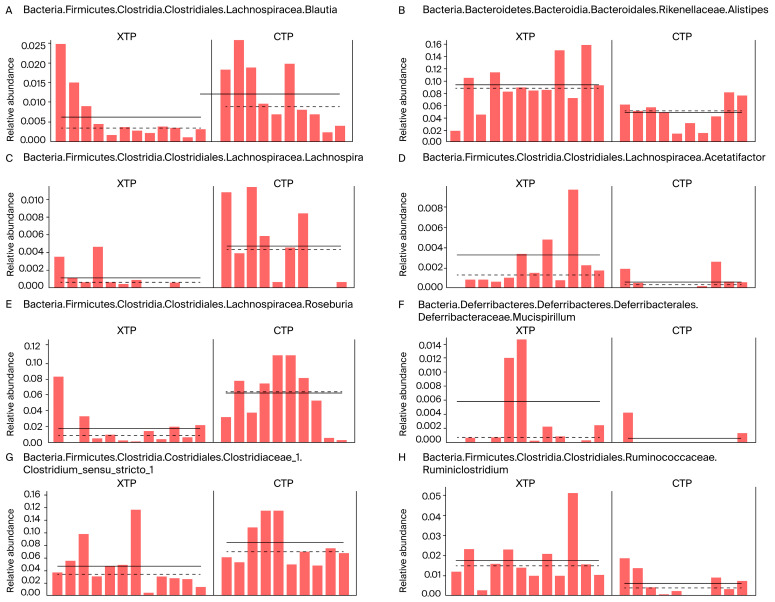
Significant changes in relative abundance of bacteria at the genus level were examined via LEfSe analysis. The left panel represented bacteria down-regulated in responses to MTX treatment (**A**,**C**,**E**,**G**). The right panel represented bacteria up-regulated in responses to MTX treatment (**B**,**D**,**F**,**H**). LDA scores higher than 3 were shown with significant changes. Mann–Whitney U test was performed to examine the significant differences between CTP and XTP groups. CTP: sham groups; XTP: MTX-treated groups. N = 10 in CTP; N = 12 in XTP.

Feces collected from rats receiving combined IT and IP MTX treatment (XTP group) and those treated with normal saline (CTP group) were analyzed for bacterial 16S rRNA sequencing. The Chao1 value and Shannon’s biodiversity index were not significantly changed, indicating that the richness of the microbiota was not affected by MTX treatment (Figure 2A). However, the beta diversity index calculated by unweighted unifraction distance was significantly decreased in response to MTX treatment (Figure 2B), and PCoA demonstrated that bacterial composition changed following MTX treatment (Figure 2C). In addition, the Firmicutes/Bacteroidetes ratio decreased in the XTP group, suggesting a propensity for inflammatory bowel diseases (Figure 2D). Moreover, the relative abundance of the top 10 clusters of bacteria at the phylum and family levels revealed an alteration in bacterial composition in response to MTX (Figure 2E,F). Linear discriminant analysis (LDA) effect size (LEfSe), which was analyzed at the genus level, showed that bacteria related to butyric acid and propionic acid production were decreased (*Roseburia*, *Blautia*, *Clostridium_sensu_stricto_1*, and *Lachnospira*), while acetic acid-producing bacteria were increased (*Acetatifactor*) (Figure 2G). Furthermore, MTX treatment stimulated the up-regulation of *Mucispirillum*, *Alistipes*, and *Ruminiclostridium* at the genus level compared to that of the CTP group (Figure 3).

### 3.2. Metabolic Pathway Alterations Following MTX Treatment

**Figure 4:**
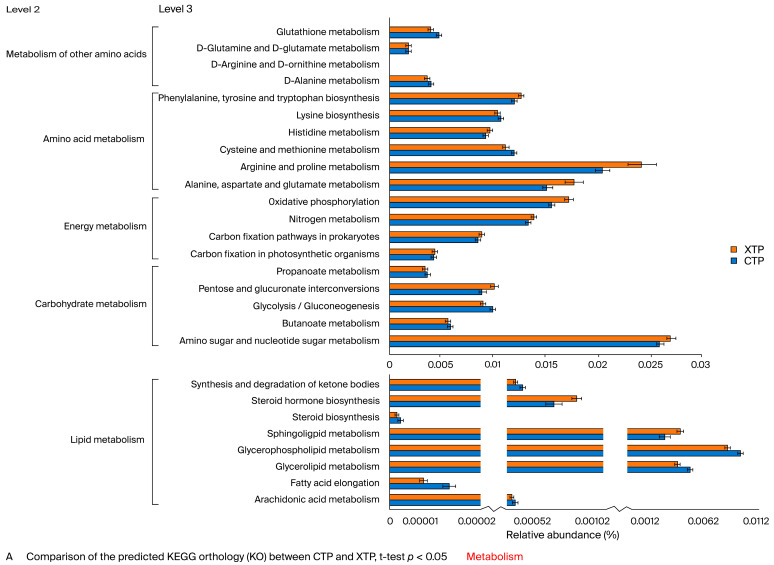

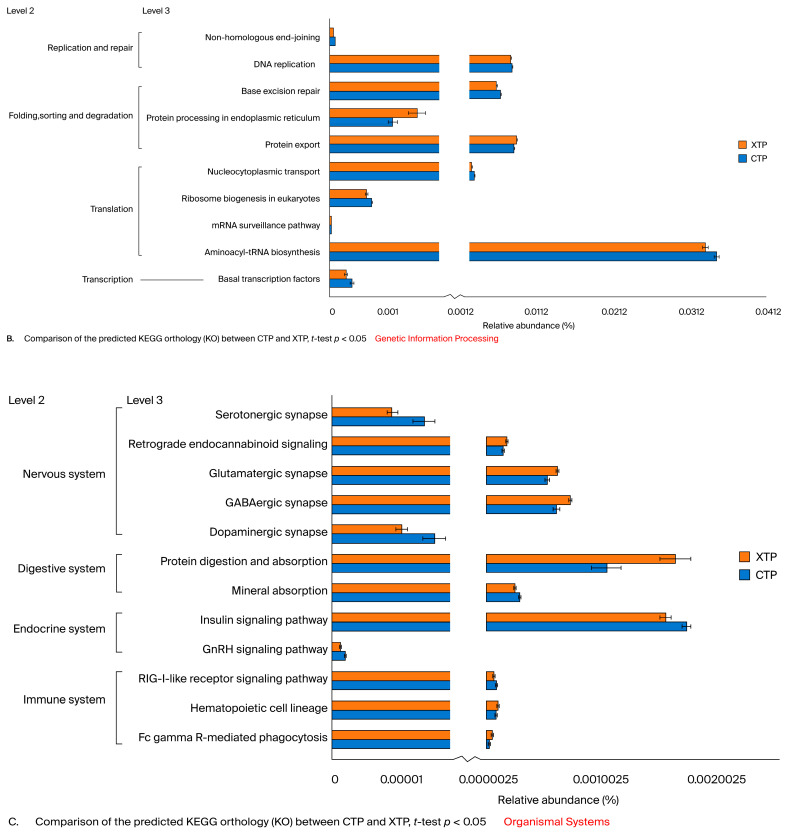
KEGG pathway under metabolism and enriched KEGG pathways in categories of metabolism (**A**), genetic information processing (**B**), and the organismal system (**C**), at classification levels 2 and 3. The bar plots show relative abundance (%). Statistical analysis was performed by independent *t*-test, *p* < 0.05.

We found that most level 2 KEGG pathways were enriched in amino acids (Figure 4A). At level 3, KEGG pathway analysis revealed the up-regulation of phenylalanine, tyrosine, tryptophan, alanine, aspartate, glutamate, arginine, and proline metabolism. An analysis of the energy metabolism pathway at level 3 showed that up-regulated oxidative phosphorylation and nitrogen metabolism were found in the XTP group. In contrast, carbohydrate metabolism in the level 3 analysis showed a decrease in glycolysis/gluconeogenesis and butanoate metabolism pathways after MTX treatment. Furthermore, most lipid metabolism under level 3 KEGG pathways was decreased in response to MTX, except for steroid hormone biosynthesis and sphingolipid metabolism.

As for the genetic information process category, MTX treatment repressed most nucleic acid processing pathways, including replication, repair, and translation/transcription. Notably, MTX treatment significantly enhanced the folding, sorting, and degradation pathways, especially protein export and protein processing in the endoplasmic reticulum (Figure 4B). Furthermore, the organismal system analysis at level 2 revealed that most enriched pathways were closely related to the nervous system, which implies a high susceptibility of the CNS in response to MTX treatment (Figure 4C).

### 3.3. SCFA and NT Metabolism Were Affected in Response to MTX Treatment

**Figure 5:**
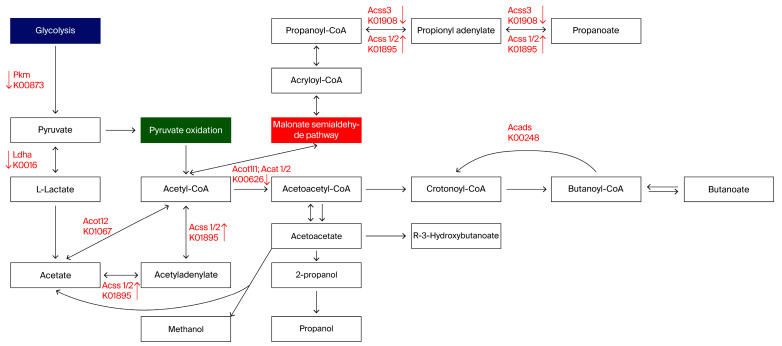
Scheme of short-chain fatty acid metabolism pathway affected by MTX. Genes highlighted in red were verified by real-time PCR followed by *t*-test statistical analysis. Up arrow indicates up-regulated genes compared to the CTP group, *p* < 0.05; down arrow shows down-regulated genes compared to the CTP group, *p* < 0.05. CTP: sham groups; XTP: MTX-treated groups. N = 8 in each group.

**Figure 6:**
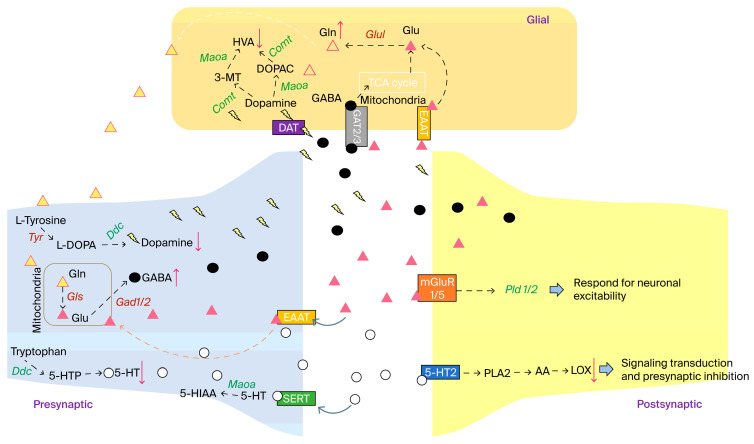
Neurotransmitter dysregulation and alteration in neurosynaptic communication following MTX treatment. The genes labeled in green and italics were down-regulated following MTX by independent *t*-test compared to the CTP group, *p* < 0.05. The genes labeled in red and italics were up-regulated following MTX by an independent *t*-test compared to the CTP group, *p* < 0.05. The red arrows with a down direction represented a decreased production of the corresponding metabolites. The red arrows with an up direction represented an increased production of the corresponding metabolites. Glul: K01915 Glutamine synthetase; Comt: K00545 Catechol O-methyltransferase; Maoa: K00274 Monoamine oxidase; Pld1/2: K01115 Phospholipase D1/2; Ddc:K01593 Aromatic-l-amino-acid/l-tryptophan decarboxylase; Gad 1/2: K01580 Glutamate decarboxylase; Gls: K01425 Glutaminase; Tyr: K00505 Tyrosinase.

The data showed that the mapped genes were highly associated with short-chain fatty acid (SCFA) and neurotransmitter (NT) metabolism pathways (Figure 5 and Figure 6).

*Acads* (K00248; butyryl-CoA dehydrogenase [EC:1.3.8.1]), *Acat 1/2* (K00626; acetyl-CoA C-acetyltransferase [EC:2.3.1.9]), *Acss 1/2* (K01895; acetyl-CoA synthetase [EC:6.2.1.1]), and *Acss 3* (K01908; propionyl-CoA synthetase [EC:6.2.1.17]) were mapped to butanoate and propionate metabolism pathways (Figure 5). Moreover, level 3 KEGG analysis of carbohydrate metabolism revealed the down-regulation of butanoate metabolism in the XTP group. These results indicate that SCFA production may be affected by MTX treatment.

The short-chain fatty acid metabolism pathway was affected by MTX; hence, the production of short-chain fatty acids may be reduced following MTX treatment. Genes highlighted in red were verified by real-time PCR. The up arrow indicated that the gene expression level was up-regulated compared to the CTP group via a *t*-test statistical analysis and *p* < 0.05; the down arrow was down-regulated compared to the CTP group via *t*-test statistical analysis and *p* < 0.05. CTP: sham groups; XTP: MTX-treated groups. N = 10 in CTP.

According to the level 3 KEGG pathway prediction of the extent of the impact of MTX treatment on the nervous system, glutamatergic synapses, GABAergic synapses, dopamine synapses, and serotonergic synapses were highly affected (Figure 4C). In addition, level 4 Tax4Fun gene enrichment showed that *pld 1/2* (K01115; phospholipase D [EC:3.1.4.4]), *gls* (K01425; glutaminase [EC:3.5.1.2]), and *glul* (K01915; glutamine synthetase [EC:6.3.1.2]) are involved in the neuronal synapses mentioned above (Figure 6). MTX resulted in the significant down-regulation of *pld 1/2* expression level at the postsynapse, while *glul* was up-regulated in glial cells. Glutaminase (*gls*) was able to transfer glutamine into glutamate at the presynapse, although it was not significantly changed by MTX. The above results indicate that the hemostasis of neuronal communication was disrupted by MTX. Moreover, the *Ddc* (K01593; aromatic-l-amino-acid decarboxylase [EC:4.1.1.28]) gene, which plays an important role in dopamine (3,4-dihydroxyphenethylamine, DA) and serotonin (5-hydroxytryptamine, 5-HT) synthesis pathways, was down-regulated in response to MTX treatment, which may result in a decrease in DA and 5-HT. In contrast, *gad 1/2* (K01580; glutamate decarboxylase [EC:4.1.1.15]) genes that utilize glutamate to synthesize GABA were up-regulated by MTX, while the *glul* expression level was also increased by MTX, which resulted in the elevation of glutamine (Gln) levels since it plays an essential role in the synthesis of glutamine from glutamate (Glu). Taken together, MTX treatment results in an alteration in NTs and the dysregulation of neuron crosstalk, and therefore it has a detrimental impact on CNS function (Figure 6).

### 3.4. MTX Results in NT Alteration in Brain Tissue and Dysregulated SCFA Concentration Levels in the Circulating System

Genes selected from the KEGG pathway via Tax4Fun at level 4 analysis were generalized into three categories: SCFAs, glutamatergic and GABAergic synapse, and neurotransmitter-related genes. From the PICRUSt prediction, there were three genes that corresponded to the regulation of SCFA production: Acads corresponds to K00248, Acat2 corresponds to K00626, and Acot12 corresponds to K01067. We examined the mRNA expression levels of these three genes in the prefrontal cortex and dorsal hippocampus to verify the effect of MTX on their regulation. As shown in Table 1, most of the mRNA expression patterns were similar in the prefrontal cortex and hippocampus, whereas discordant changes in mRNA expression were noted in Acads, Acat1, Ldha, and Pkm. Moreover, significant changes in Acat 1, Acot12, Acss1, and Pkm mRNA expression in both the prefrontal cortex and hippocampus and a significant decrease in the mRNA expression of hippocampal Acat2 and cortex Acss 3 RNA were noted (Table 1).

Genes associated with glutamatergic synapses and GABAergic synapses selected from PICRUSt predictions were verified in brain tissue, and a significant decrease in *Pld1* mRNA expression was found in the cortex, with a significant increase in *Gls* and *Gls 2* mRNA expression in the cortex. Notably, a significant increase in *Glu1* mRNA expression was noted in both the prefrontal cortex and hippocampus. Furthermore, we verified the genes that were closely related to NT regulation and found significant increases in *Ggt6*, *Tyr*, *Gad1*, and *Gad2* mRNA expression in the brain cortex and decreased *Ggt6*, *Gad1*, and *Maoa mRNA* expression in the hippocampus following MTX treatment. In addition, decreased Comt mRNA expression was observed in the cortex of rats in the XTP group (Table 1).

### 3.5. A Strong Relationship between Gut Microbiota Changes and MTX Neurotoxicity

**Figure 7:**
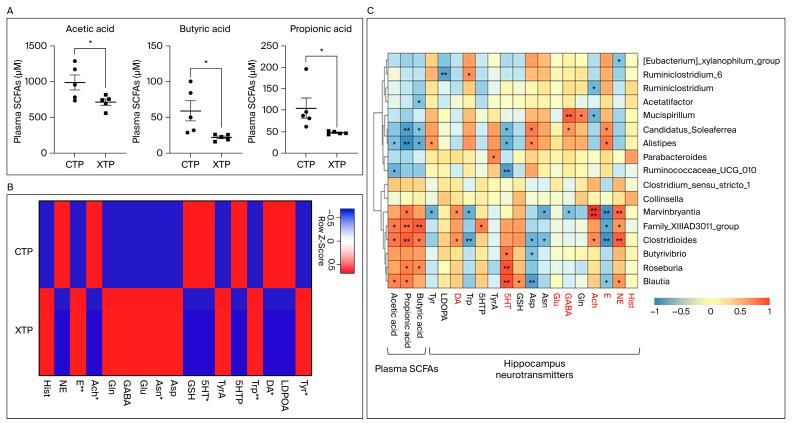
Levels of plasma SCFA and hippocampus NT metabolites were highly affected by MTX treatment. (**A**) Plasma levels of SCFAs including acetic acid, butyric acid, and propionic acid were significantly reduced in MTX groups. (**B**) Changes in 17 NT metabolites in the hippocampus after MTX treatment, analyzed by UHPLC-MS/MS, are shown in the heat map. Values were calculated as the z-score and an independent *t*-test analysis was performed. *: compared to CTP, *p* < 0.05; **: compared to CTP, *p* < 0.01. (**C**) Correlations between genus and metabolites in the plasma and hippocampus. The 17 bacteria, on the genus level, were selected in metagenomeSeq data. Spearman’s coefficients were indicated by a color gradient from blue (negative correlation) to red (positive correlation). *: *p* < 0.05; **: *p* < 0.01. Tyr: l-Tyrosine; LDOPA: 3,4-Dihydroxyphenylalanine; DA: dopamine; Trp: l-Tryptophan; 5HTP: 5-Hydroxytryptophan; TrpA: Tryptamine; 5HT: Serotonin; GSH: Glutathione; Asp: l-Aspartate; Asn: l-Asparagine; Glu: l-Glutamate; GABA: 4-Aminobutyric acid; Gln: l-Glutamine; Ach: Acetylcholine; E: Epinephrine; NE: Norepinephrine; Hist: Histamine. Metabolites shown in red were associated with learning and memory function in the brain.

To further evaluate the effect of MTX on plasma SCFA regulation, we used GC-MS to determine the plasma levels of SCFAs. As shown in Figure 7A, plasma levels of SCFAs decreased after MTX treatment. In addition, the levels of acetic acid (F = 5.452, *p* < 0.05) and butyric acid (F = 14.192, *p* < 0.01) were mostly affected by MTX treatment, which is consistent with the dysbiosis of microbiomes at the family level of bacteria belonging to Clostridia clusters IV (Ruminococcaceae) and XIVa (Lachnospiraceae). Moreover, a significant decrease in butyric acid levels was consistent with the results showing that butyric acid-producing bacteria were depleted from the fecal 16S NGS analysis (Figure 7C).

The metabolite analysis of the metabolome revealed a significant decrease in serotonin, dopamine, and acetylcholine (Ach) in the hippocampus following MTX treatment. MTX reduced the levels of norepinephrine (NE), but the differences were not significant. In contrast, epinephrine (E) significantly increased in response to MTX, and similar patterns were observed for l-glutamic acid (Glu) and 4-aminobutyric acid (GABA). However, histamine (Hist) levels were not significantly altered by MTX (Figure 7B). These results were consistent with previous predictions based on KEGG pathway enrichment.

As mentioned above, MTX treatment significantly changed the levels of plasma SCFAs (Figure 7A) and hippocampal NT metabolites (Figure 7B). Correlations between the alterations in microbiota at the genus level and metabolites in the plasma and hippocampus were analyzed. Seventeen bacteria at the genus level were selected from the metagenomeSeq data. As shown in Figure 7C, we found a strong relationship between gut microbiota changes and alterations in metabolites, which implies that MTX treatment alters the gut microbiota and provides clues about the pathogenesis of MTX neurotoxicity.

## 4. Discussion

The main findings of this study are summarized as follows. First, MTX treatment resulted in gut microbiota changes, as evidenced by alterations in the relative abundance of the microbiome at the phylum, class, order, family, and genus levels compared to that of the control group. Second, Tax4Fun level 4 annotation genes used to map with the KEGG pathway mapper website found that the mapped genes were highly associated with SCFA and NT metabolism pathways. Third, glutamatergic and GABAergic synapse-related gene expression in the prefrontal cortex and hippocampus changed and NT-regulation gene expression in the hippocampus was altered after MTX treatment. Fourth, levels of plasma SCFAs and hippocampus NT metabolites were significantly affected by MTX treatment. Fifth, we found a strong relationship between gut microbiota changes and alterations in gut and brain metabolites (Figure 7C), which implies that MTX treatment alters the gut microbiota and provides clues about the pathogenesis of MTX neurotoxicity.

In this first part of the study, we found that the bacteria associated with SCFA-producing bacteria (especially butyric acid and propionic acid), such as *Roseburia*, *Blautia*, *lostridium_sensu_stricto_1*, and *Lachnospira*, were decreased at the genus level, while *Acetatifactor,* bacteria that produce acetic acid, were increased following MTX treatment. In addition, bacteria such as *Mucispirillum* and *Alistipes*, which were reported to be related to various human neurologic disorders such as Alzheimer’s disease and major depressive disorder, increased markedly after MTX treatment [50,51]. To clarify the impact of gut microbiota changes on the nervous system following MTX treatment, we used the KEGG pathway enrichments analyzed by PICRUSt and found that butanoate and most of the lipid metabolism pathways were down-regulated in response to MTX, which was consistent with the reduced plasma levels of SCFAs found in our study. In addition, we examined the SCFA-related genes in the prefrontal cortex and hippocampus and found altered *Acat 1*, *Acot 12*, *Acss1,* and *Pkm* expression in both the prefrontal cortex and hippocampus, and changes in *Acss 3* in the prefrontal cortex and *Acat2* in the hippocampus. Since the hippocampus plays an essential role in learning and memory function, and owing to limited brain specimens, we performed neurotransmitter metabolite analysis via GC/MS detection only in the hippocampus and found that the neurotransmitters that changed after MTX treatment were associated with learning and memory processes. Taken together, these findings suggested that combined IT and IP MTX treatment resulted in gut microbiota changes, which might be a contributing factor resulting in neurologic dysfunction following MTX treatment.

To ascertain the effect of MTX on gut microbiota changes and its interaction with neurogenesis, we examined glutamatergic and GABAergic synapse-related gene expression in the prefrontal cortex and hippocampus via real-time PCR, mainly focusing on the four genes selected from PICRUSt predictions for their roles in neuron repair and neurogenesis, and we found that MTX resulted in significant changes in *pld 1/2*, *glul*, and *Ddc* mRNA expression. Phospholipase D (*pld*), one kind of phosphatidic acid, has been implicated in neurotransmission and mainly implicated in pre- and postsynapse neuron function [52,53]. Similarly, glutaminase (*gls*) plays an important role through its ability to transfer glutamine into glutamate at the presynapse [54]. In addition, the *gls* gene plays an important role in the maturation of neuron progenitor cells [55]. Moreover, the *Ddc* gene, which plays an important role in the DA and 5-HT synthesis pathways and is crucial for normal brain development [56,57], was found to be down-regulated in response to MTX treatment, which may result in a decrease in DA and 5-HT. In contrast, *gad 1/2* (K01580; glutamate decarboxylase [EC:4.1.1.15]) genes that utilize glutamate to synthesize GABA [58,59] were up-regulated by MTX, while *glul* expression level was also increased by MTX, which may elevate the glutamine (*Gln*) levels because it plays a role in the synthesis of glutamine from glutamate (*Glu*). The above results indicate that the hemostasis of neuronal communication was disrupted by MTX treatment. Therefore, we assumed that the development of MTX neurotoxicity may have resulted from the dysregulation of the NT transmission process.

SCFAs are metabolites produced in the colon by the bacterial fermentation of dietary fibers and resistant starch [60,61]. Growing evidence supports the idea that SCFAs exert crucial physiological effects on the brain, which is supported by studies in animals and humans showing that gut microbiota changes have been implicated in behavioral and neurological pathologies, such as depression, Alzheimer’s disease (AD), Parkinson’s disease (PD), and autism spectrum disorder (ASD) [62,63,64,65]. Manipulation of the gut microbiota and SCFAs have been proposed as treatment targets for various neurological disorders [66]. Furthermore, increasing evidence suggests that the composition of the gut microbiota is related to MTX neurotoxicity, and microbial function is closely linked to MTX neurotoxicity. In this study, it is worth noting that circulating levels of SCFAs and NTs were related to a microbial signature (Figure 7C), which suggests that MTX neurotoxicity is closely associated with the gut microbiota change induced by MTX treatment.

Recent studies have indicated that the modulation of gut microbiota is a way to reduce MTX GI toxicity [17,18,67,68], with an emphasis on the impact of MTX on gut microbiota homeostasis. However, prior studies on MTX toxicity have mostly focused on MTX intestinal toxicity and gut environment changes, emphasizing taxonomic composition based on 16S rRNA sequencing [17,69]. Despite analysis beyond taxonomic composition to analyze microbial functionality, metabolomics, and shotgun metagenomics, sequencing is vital to accurately capture host and microbiome interplay, and a comprehensive study on MTX toxicity beyond taxonomic composition is lacking. Our previous study revealed that combined IT and IP MTX treatment resulted in spatial memory changes in developing rats; therefore, we tried to explore gut microbiota changes and to determine if the interruption of normal gut–brain axis communication is elicited by MTX in this study. This may present a candidate for a preventive and therapeutic strategy for MTX neurotoxicity by manipulating the gut microbiota.

Unlike previous studies dealing with MTX toxicities, our study discusses MTX neurotoxicity, not only by exploring the gut microbiota composition but also by discussing metabolome changes following MTX treatment, focusing on the interaction between NTs and SCFA alteration and gut microbiota change. Furthermore, this is the first study utilizing a young rat model to explore the impact of MTX on the gut and brain, which provides a model of chemobrain in developing rats. Truely, the impact of this study in relevance to clinical application lies in its role in providing insight into personalized medical treatment designation. Notably, our study is the first one to comprehensively evaluate gut microbiota changes following MTX treatment and to further evaluate SCFA and NT alterations to determine the role of the gut–brain axis in the pathogenesis of MTX neurotoxicity. In clinical settings, the findings of this study suggest a method for personalized medical care that is based on gut microbiota changes in order to adjust therapeutic medications in the future.

Our study had several limitations. First, we did not measure fecal SCFA concentrations but used plasma SCFAs owing to the limited fecal specimens, and we found unparallel changes in the gut microbiota and plasma SCFAs. Since unparallel changes in plasma and fecal SCFA concentrations have been reported in various neurological disorders and medical interventions [70,71], it is mandatory to check fecal SCFA concentrations in further studies to create a more comprehensive view of gut–brain crosstalk following MTX treatment. Second, only the hippocampal and prefrontal cortex mRNA expression levels of SCFA-related and neuron-repair genes were measured. Since the study highlights the interaction between gut microbiota change and SCFA/neurotransmitter expression following MTX treatment, which can be explained by the correlation between the alterations in microbiota at the genus level and metabolites in the plasma and hippocampus (Figure 7C), we did not directly measure the protein expression of the individual genes examined in the study because of limited specimens. For a better explanation of the gut–brain communication in the MTX-treated young rat model, a specific examination of the individual genes involved in SCFA and neuron repairment is crucial in following studies. Third, since this is an extension study of our previous work, based on the findings that combined IT and IP treatment result in cognition impairment, we only focus on gut microbiota change in young rats receiving combined IT and IP treatment. To better understand the impact of MTX treatment on gut microbiota under different routes of MTX administration, further studies to specifically group animals into IP, IT, and combined IT and IP groups are required.

In our previous study, we found a direct effect of MTX on the nervous system in developing young rats, particularly regarding cognitive function [13,14]. In this work, we found gut microbiota changes with relevant alterations in SCFA/NT gene regulation, which suggest that the gut–brain axis plays an essential role in MTX neurotoxicity. To our knowledge, this is the first study to explore the interaction between gut microbiota changes and neurotransmitter alterations in developing rats following MTX treatment. These findings shed light on the prevention and intervention of MTX neurotoxicity, mainly through the manipulation of gut microbiota.

## Figures and Tables

**Table 1:** Verification of expression for genes responsible for SCFA and neurotransmitter production in cortex and hippocampus. mRNA levels were verified in cortex and hippocampus (Hippo) tissues. Direction of arrow up or down shows up-regulation or down-regulation, respectively, in the XTP group compared to the CTP group. An independent *t*-test was used to test the significant differences between groups. Significance of expression change: —non-significant; ↓/↑—significant, *p* < 0.05; ↓↓/↑↑—significant, *p* < 0.01. CTP: sham group; XTP: MTX-treated group. N = 8 in each group.

	Predicted Pattern	Verified Genes	Cortex	Hippo
	(XTP vs. CTP)		(XTP vs. CTP)	(XTP vs. CTP)
**SCFAs Gene**				
*Acads*	-	*Acads*	-	-
*Acat*	↓	*Acat1*	↑↑	↓
		*Acat2*	-	↓↓
*Acot12*	-	*Acot12*	↑	↑
*Acss1/2*	↑	*Acss1*	↑	↑
		*Acss2*	-	-
*Acss3*	↓	*Acss3*	↓	-
*Ldha*	↓↓	*Ldha*	-	-
*Pkm*	↓	*Pkm*	↑↑	↓
**Glutamatergic ** **and GABAergic synapse genes**				
*Pld1/2*	↓	*Pld1*	↓	-
		*Pld2*	-	-
*Gls*	-	*Gls*	↑	-
		*Gls2*	↑	-
*Glul*	↑	*Glul*	↑	↑↑
**Neurotransmitter genes**				
*Tyr*	↓	*Tyr*	↑↑	-
*Ddc*	↓↓	*Ddc*	-	-
*Ggt6/7*	↓	*Ggt6*	↑	↓
		*Ggt7*	-	-
*Gad1/2*	↑	*Gad1*	↑↑	↓↓
		*Gad2*	↑	-
*Glul*	↑	*Glul*	↑	↑↑
*Comt*	↓	*Comt*	↓	-
*Maoa*	↓	*Maoa*	-	↓

## Data Availability

We appreciate C.-Y.H. for the contribution in analysis of the metabolome and microbiome. The data that support the findings of this study are available from the corresponding author upon reasonable request.

## Supplementary Materials

The following supporting information can be downloaded at https://www.mdpi.com/article/10.3390/biomedicines12040908/s1, Table S1. Sequences of the genes examined in this study; Figure S1. Verification of SCFA-related gene expression levels in the hippocampus and cortex; Figure S2. Verification of glutamatergic synapse and GABAergic synapse correlation with gene expression levels in the hippocampus and cortex; Figure S3. Verification of neurotransmitter (NT) correlation gene expression levels in the hippocampus and cortex.
