# Supplementary material for: The Impact of Gut Microbiota Changes on Methotrexate-Induced Neurotoxicity in Developing Young Rats
Source: Biomedicines. 2024 Apr 19;12(4):908. doi: 10.3390/biomedicines12040908 (PMC11048417; doi:10.3390/biomedicines12040908)
Supplement: Supplementary file 1 [file biomedicines-12-00908-s001.zip › biomedicines-2936219-supplementary.pdf]

Supplementary Materials

Table S1. Sequences of the genes examined in this study.

**SCFAs pathway genes**		
**Genes**	**Primer F 5'->3'**	**Primer R 5'->3'**
ACADS	*ATGCCAACTCCTGGGCTTAC*	*GGGATGCGACAGTCCTCAAA*
Acat1	*CAGGTCTACCCATTGCCACT*	*ACCGTATGGTGTTGCTCCTC*
Acat2	*TGTCATGGGAGTAGGACCGA*	*ACTGCTGCAAAGGCTTCATT*
Acot12	*ACCTGCTCGGTGGTAAATGG*	*CGTCCTTGAGGGGCTTTCTT*
Acss1	*ACCCTGATGCTGGTCGTTAC*	*CCTCATGATTGATGGGCTCT*
Acss2	*GAACATGAGGCTGTTGCAGA*	*TCTGAGCAATCTTCCGGAGT*
Acss3	*GTACAAGCCCTGGACCAAAA*	*CACCAGCCAGCTTAGAGACC*
Pkm	*ATGAATGTGGCTCGGCTGAA*	*GCCCTTGATGAGTCCAGTCC*
Ldha	*CAGTGCAAACTGCTCATCGT*	*CGACATTCACACCACTCCAC*
**Glutamatergic and GABAergic synapse pathway genes**		
Pld1	*CAACTCGGACAGCATTAGCA*	*CGTGAACCACAGAACCAATG*
Pld2	*GAGAGCTCCATCCTCAATGC*	*GAAACACTGCCCCTGTTCAT*
Gls	*CTCCGAAGGTTTGCTCTGTC*	*CATCATGGTGTCCAAAGTGC*
Gls2	*GAGGATGCCAAAGAGCTCAC*	*TAGTCGGTGCCTAAGGTGCT*
Glul	*AAGCTGGTGTTCTGCGAAGT*	*GCCATAAGCCTTGTCAGCTC*
**NTs pathway genes**		
Ggt6	*GCTCCTTTGGCTCTGGACAT*	*TTGGACCTGCTGTTGGGTTT*
Ggt7	*ACTTTTGCCACGGGTGTTAC*	*TGGAGCCACTATTCCCAAAC*
Tyr	*GCTTGGGGGCTCTGAAATCT*	*GACGCTGGGCTGAGTAAGTT*
Ddc	*CAGCTCCTACCCAGCTATGC*	*TTTTAGTCCGAGCAGCCAGT*
Gad1	*CCAACCTGCGTCCTACAACA*	*TCAGGCCCAGTTTTCTGGTG*
Gad2	*CTGTGTACGGGGCTTTTGAT*	*TGCATCAGTCCCTCCTCTCT*
Maoa	*AGCAAGACACGCTCAGGAAT*	*TGCCTCACATACCACAGGAA*
Comt	*CCCTGACTTCCTGGCGTATG*	*TGGACCCTGGTAGATTGCCT*

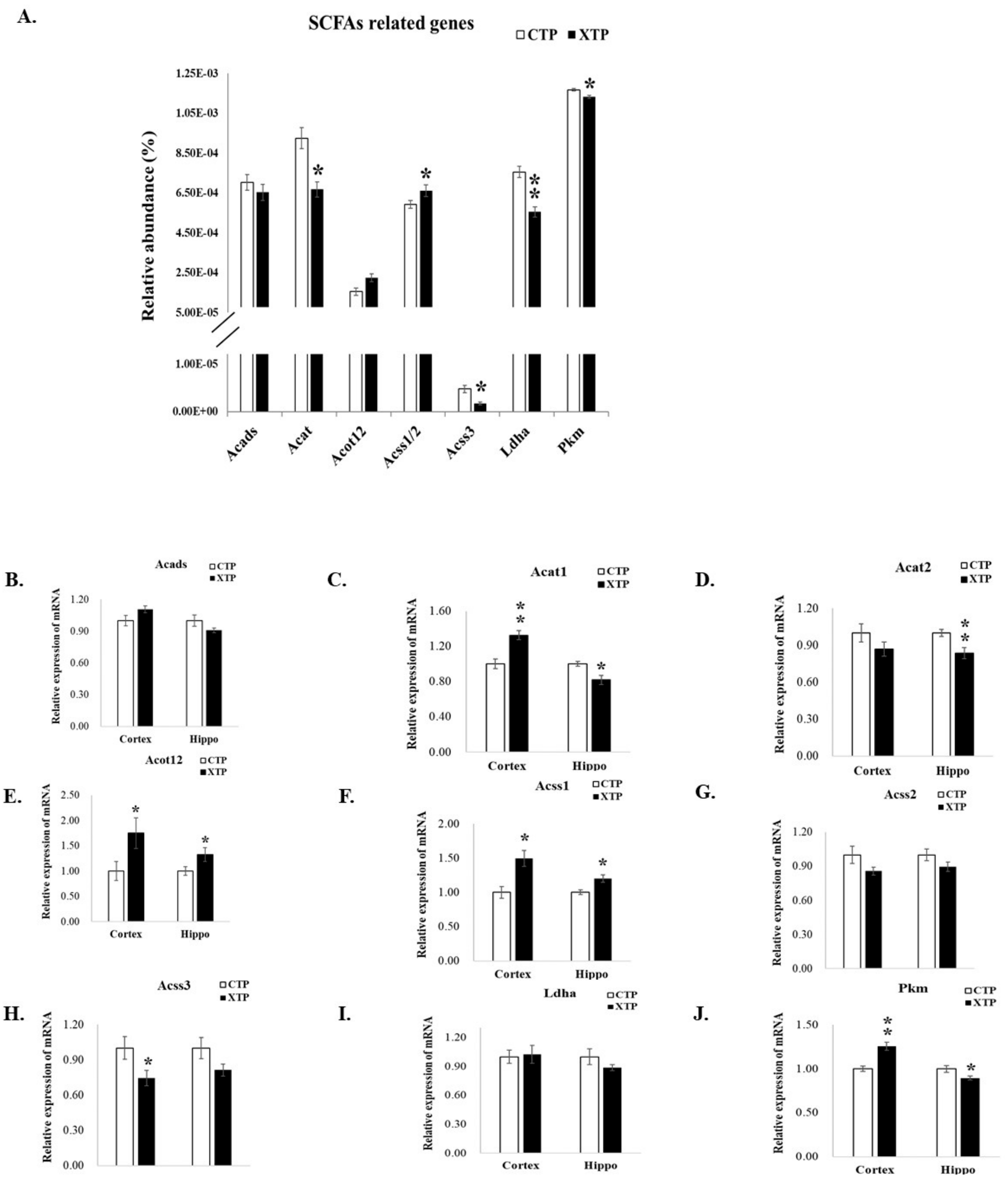

Figure S1. Verification of SCFA-related gene expression levels in the hippocampus and cortex. (A) Specific genes correlated with SCFA metabolism were selected from the enrichment of KEGG pathways via Tax4Fun at level 4 analysis with gene expression, which were shown via significant differences (*t*-test, $p < 0.05$). Hippocampus and cortex mRNA expression levels at *Acads*(B), *Acat 1*(C), *Acat 2* (D), *Acot 12*(E), *Acss 1*(F), *Acss 2* (G), *Acss 3*(H), *Ldah* (I), and *Pkm* (J) were examined via real-time PCR. Values were shown in fold changes compared to the CTP group, and the statistical analysis was performed by an independent *t*-test. CTP: sham groups; XTP: MTX-treated groups. *: $p < 0.05$ vs. CTP; ** $p < 0.01$ vs. CTP . N = 8 in cortex and hippocampus in each group.

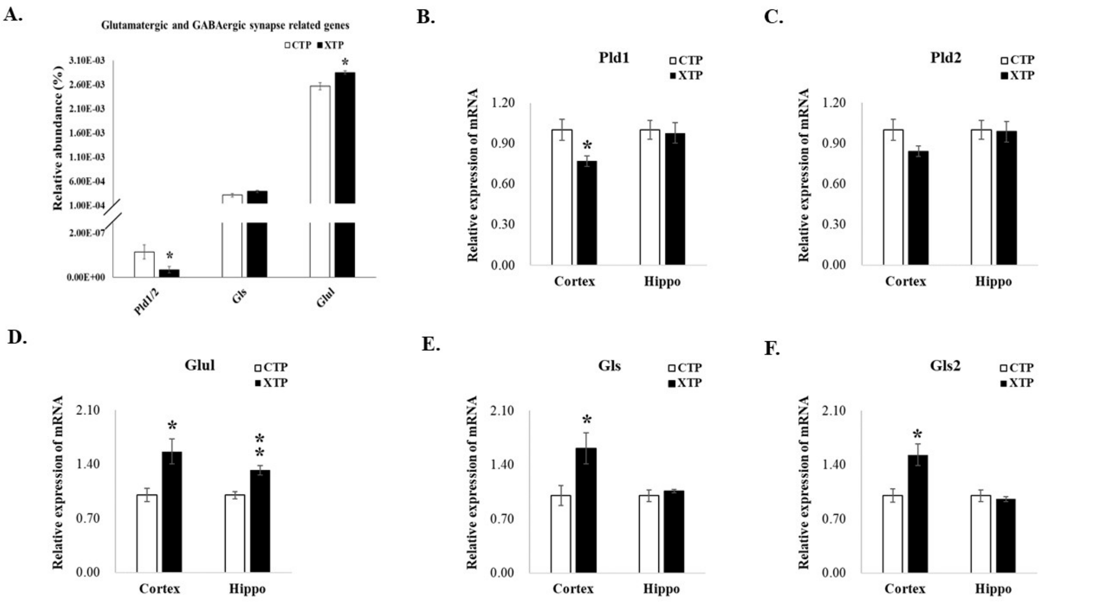

Figure S2. Verification of glutamatergic synapse and GABAergic synapse correlation with gene expression levels in the hippocampus and cortex. (A) Specific genes correlated with synapses were selected from the enrichment of KEGG pathways via Tax4Fun at level 4 analysis with gene expression showing significant differences (t-test, $p < 0.05$). Hippocampus and cortex mRNA expression levels at *Pld 1*(B), *Pld 2* (C), *Glul* (D), *Gls* (E), and *Gls 2* (F) were examined via real-time PCR. Values were shown in fold changes compared to the CTP group, and the statistical analysis was performed by an independent t-test. CTP: sham groups; XTP: MTX-treated groups.*: $p < 0.05$ vs. CTP; ** $p < 0.01$ vs. CTP. N = 8 in cortex and hippocampus in each group.

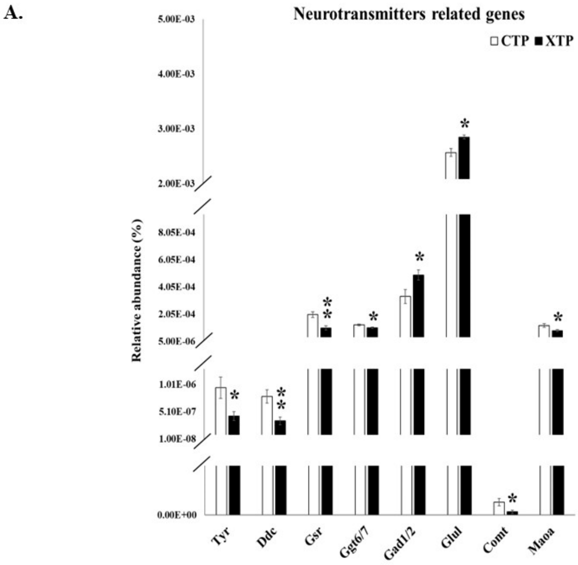

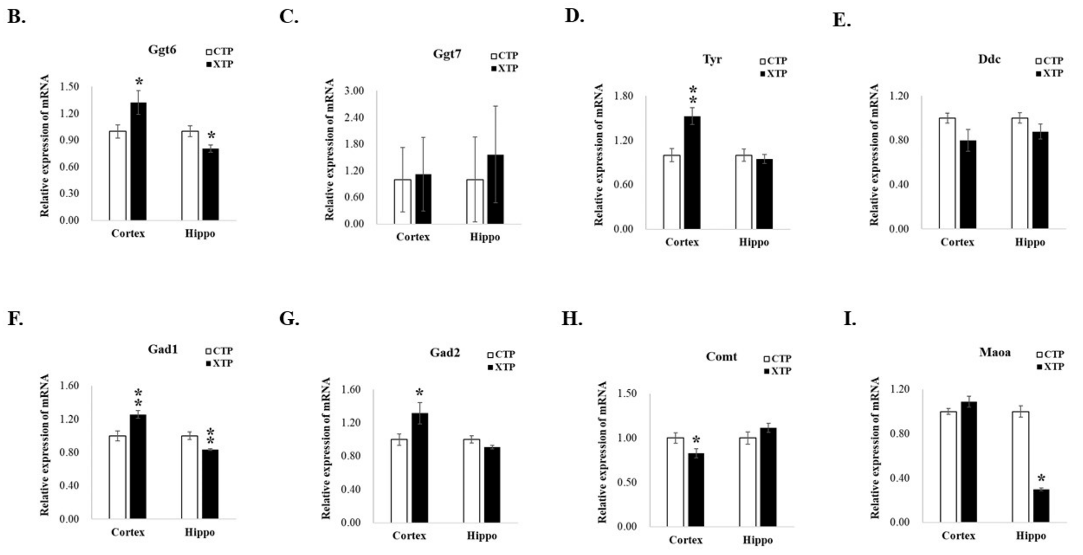

Figure S3. Verification of neurotransmitter (NT) correlation gene expression levels in the hippocampus and cortex. (A) Specific genes correlated with NTs were selected from the enrichment of KEGG pathways via Tax4Fun at level 4 analysis with gene expression showing significant differences (t-test, $p < 0.05$). Hippocampus and cortex mRNA expression levels at *Ggt6* (B), *Ggt7*(C), *Tyr*(D), *Ddc* (E) , *Gad1*(F), *Gad2* (G), *Comt* (H), and *Maoa* (I) were examined via real-time PCR. Values are shown in fold changes compared to the CTP group, and the statistical analysis was performed by independent t-test. CTP: sham groups; XTP: MTX-treated groups.*: $p < 0.05$ vs. CTP; ** $p < 0.01$ vs. CTP. N = 8 in cortex and hippocampus in each group.
